# Conditional superiorities and unaddressed bottlenecks: a critical review of artificial intelligence for waterborne microbial detection

**DOI:** 10.1007/s00253-026-13917-8

**Published:** 2026-07-17

**Authors:** Weiqi Liu, Qiang Tang, Min Shen, Lin Zhang, Xu Jia

**Affiliations:** 1 Key Laboratory of Non-Coding RNA, Drug Discovery at Chengdu Medical College of Sichuan Province, School of Basic Medical Sciences, Chengdu Medical College, Chengdu, 610500 Sichuan China; 2 Department of Pharmacy, Shaoxing People’s Hospital, Shaoxing Hospital, Zhejiang University School of Medicine, Shaoxing, Zhejiang 312000 China

**Keywords:** Aquatic quality monitoring, AI, Pathogen detection, Environmental microbiology, Biosensor

## Abstract

**Abstract:**

Although conventional microbial detection approaches for water samples are widely applied, they still suffer from prolonged assay durations (24–72 h), low sensitivity, and the absence of real-time monitoring capacity. Artificial intelligence (AI) has demonstrated conditional advantages in specific experimental environments, such as achieving a sensitivity of 99% for detecting Cryptosporidium and Giardia in low turbidity water (based on approximately 12,000 annotated images, using fivefold cross validation, completed under laboratory conditions); however, such advantages tend to diminish or vanish in high-turbidity water matrices or when training datasets are insufficient. This review critically evaluates four categories of AI-driven approaches: image-based analysis, spectroscopic techniques, genome, and metagenomic sequencing, as well as predictive pollution modeling. While AI helps boost detection efficiency, precision, and analytical capacity, a set of long-standing obstacles restrict its real-world deployment. The main issues involve non-standardized datasets, low model interpretability, weak generalization over various water substrates, and a substantial gap between lab-based performance and on-site operational outcomes. In summary, to fully exploit the capabilities of AI in aquatic microbial detection, greater emphasis should be placed on on-site validation, unified data specifications, and practical performance benchmarks, rather than further algorithmic innovation. This review seeks to provide practical references for scholars and practitioners working in the fields of microbiology, AI and water quality monitoring and management.

**Key points:**

*• AI shows favorable performance for microbial detection under lab conditions*.

*• Model performance declines greatly in complex water with many practical barriers*.

*• Standardized data and validation will advance real-world application*.

## Introduction

Bacteria, archaea, algae, and protozoa are key microbial taxa that form an integral part of aquatic ecosystems (Strom 2008). However, considerable interspecific diversity and dynamic changes across time and space present considerable difficulties for reliable detection and quantification.

Although conventional cultivation approaches serve as cornerstone techniques in microbiology, they suffer from several inherent limitations: lengthy incubation, poor sensitivity, intensive manual workload, and the inability to recover viable but non-culturable (VBNC) microbes (Ramamurthy et al. 2014). Such inherent limitations greatly reduce their feasibility for real-time, high-throughput analysis, which is indispensable to modern water quality monitoring.

**Fig. 1:**
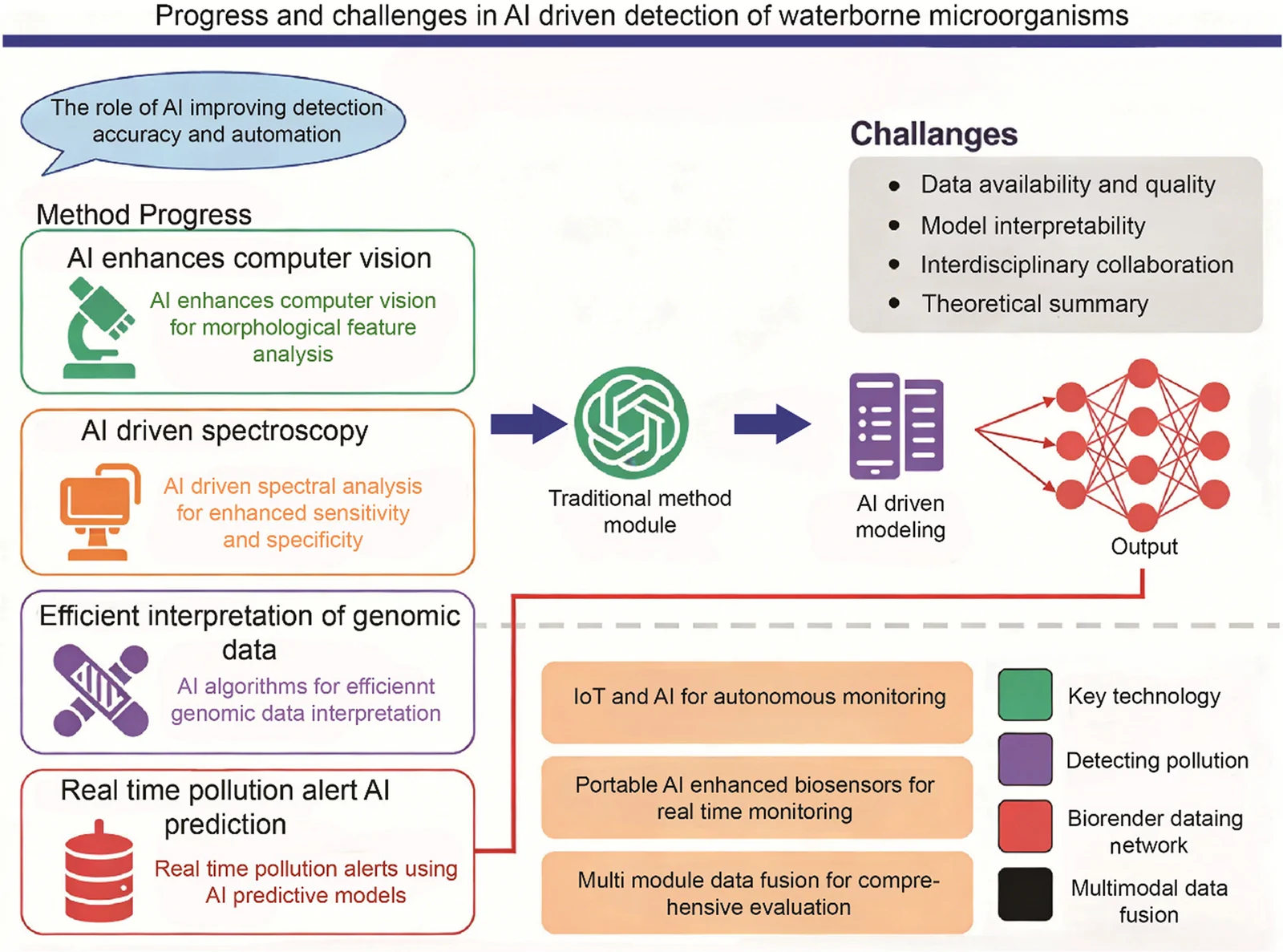
This framework encompasses four core technical pathways

Rapid progress in AI technology has triggered a fundamental paradigm shift for the detection of microorganisms in water environments (Oon et al. 2023). To illustrate the evolutionary trajectory of AI-enabled microbial detection technologies, we divide recent progress into four principal methodological groups (Fig. 1). By integrating imaging, spectral, and genomic datasets, machine learning and deep learning frameworks have enhanced the efficiency, accuracy and automation of aquatic microbial analysis (Huang et al. 2023; Yi et al. 2023). Such advances enable the transition from one-dimensional monitoring to multi-dimensional real-time surveillance. However, such performance gains are achieved only under specific conditions, including low water turbidity, distinct microbial morphology, and abundant training datasets (Zhang et al. 2024a, b, c). These favorable properties tend to diminish or even vanish in challenging scenarios such as high-turbidity water (Shi et al. 2026). Accordingly, the strengths of AI are environment-dependent rather than universally applicable. Even so, current technological advances have laid a solid foundation for integrating microbial detection into large-scale public health monitoring systems.

Although previous reviews have systematically summarized the applications of AI in aquatic microbiology, most studies predominantly focus on the technical successes achieved under controlled laboratory conditions (Oon et al. 2023). Nevertheless, limited attention has been paid to the conditional superiority of AI techniques, and few studies have quantitatively characterized the performance discrepancy between laboratory-based detection and field-scale application. Furthermore, critical barriers restricting practical deployment, including non-standardized data specifications, insufficient model interpretability, and poor generalization across diverse water matrices, remain under-explored. The difference between this review is that (1) believe that the advantages of AI depend on the environment, rather than universal advantages; (ii) identify the recurring disconnect between controlled experiments and field applicability in imaging, spectroscopy, genomics, and predictive modeling; and (iii) propose a comprehensive framework to bridge these gaps. Furthermore, this review discusses the prospective applications of emerging technologies, including self-supervised learning, transfer learning, and edge computing, aiming to enhance the intelligence and practicality of aquatic microbial detection systems. Ultimately, this review aims to facilitate real-world evaluation and accelerate the technological transformation of AI-driven aquatic ecological security monitoring.

Before systematically elaborating on the aforementioned framework and diverse technical approaches, it is essential to clarify the criteria for interpreting and citing performance data adopted in this review.

### Description of performance indicators adopted in this review

This review summarizes mainstream literature data regarding model accuracy, sensitivity, and detection limits. When interpreting these quantitative indicators, readers should recognize three critical contextual factors that substantially affect the comparability of reported research results:

1. Dataset characteristics: training datasets for AI models are derived from diverse sources, including public benchmark databases (e.g., the Global Water Pathogen Project), private laboratory datasets, and field-collected samples. These datasets vary substantially in scale, class balance, and annotation specifications, all of which exert a notable impact on model performance and should be clarified whenever available in relevant studies.
2. Water matrix conditions: the majority of reported model performances are acquired under controlled laboratory conditions, including sterile buffer solutions, deionized water, and water samples with turbidity below 5 NTU. Nevertheless, as elaborated in the following sections, the detection efficacy of identical methods inevitably degrades when deployed in complex scenarios, such as turbid surface water, wastewater, and high-flow aquatic environments.
3. Validation scheme: performance claims may come from k-fold cross validation, preservation validation, or independent external validation, each providing different generality.

In addition to dataset features, water matrix conditions, and validation experiments, dataset bias constitutes another key but insufficiently discussed background factor. Such bias substantially undermines the universal applicability of AI-driven microbial detection methods across all technical categories covered in this review, including imaging analysis, spectroscopy, genomics, and predictive modeling. The two main forms of deviation are interrelated. Geographic bias typically arises when training data are sampled from a restricted range of regions or watersheds (Gao et al. 2023). Such localized datasets fail to fully represent the broader diversity of microbial communities and heterogeneous environmental conditions in practical scenarios (Akinwole et al. 2021). Temporal bias arises when samples are disproportionately collected from dry seasons rather than rainy seasons. Spatial bias results from preferential sampling at geographically accessible sites. Classification bias refers to the insufficient coverage of rare yet clinically significant pathogens within datasets. Collectively, these biases constrain model generalization performance. Rather than inducing uniform and quantifiable performance declines, they elevate the risk of spurious correlations tied to specific sampling environments, causing models to prioritize environment-specific noise over robust biological and physicochemical features (Akinwole et al. 2021; Mahajna et al. 2022). Existing literature seldom reports or systematically assesses these inherent biases, leaving their practical impacts on cross-region and cross-scenario model deployment largely underexplored. Therefore, readers should understand that high accuracy on geographically or time-limited datasets does not guarantee equivalent performance under different conditions when explaining the performance indicators of the report.

## Methodological advances of AI in aquatic microbial detection

This section elaborates four mainstream technical approaches for aquatic microbial detection, including imaging analysis (Li et al. 2016), spectroscopy (Zhang et al. 2024a, b, c), genomics (Bentzon-Tilia et al. 2015), and predictive modeling (Mahloch 1974). These methodologies target distinct detection scenarios and research objectives, with their respective advantages highly contingent on practical environmental and experimental contexts. Imaging-based techniques are well-suited for on-site morphological screening of microorganisms, particularly in low-turbidity water environments where microbial morphological features are clearly distinguishable (La Spina et al. 2019). However, their ability to distinguish closely related species or detect VBNC status is limited (La Spina et al. 2019). In comparison, spectroscopic techniques yield molecular-level biochemical signatures for label-free microbial identification (Lu et al. 2026). Under well-controlled experimental conditions, these approaches can achieve substantially higher detection specificity than imaging-based methods (Wang et al. 2017). Nevertheless, the detection performance of spectroscopic methods is highly susceptible to water matrix interference, such as humic acid and suspended solids (Lu et al. 2026). Additionally, inconsistent matrix preparations across different scenarios pose considerable challenges to signal reproducibility. Genomic and metagenomic sequencing enable in-depth taxonomic classification and functional profiling of aquatic microorganisms, supporting precise strain-level identification and antibiotic resistance gene characterization (Olsen and Riber 2025). Nevertheless, such analytical superiority comes with inherent trade-offs, including high operational costs, lengthy turnaround times ranging from hours to days, and inherent incompatibility with real-time monitoring scenarios (Urban et al. 2021). Predictive modeling is fundamentally different from the three detection focused models. It leverages environmental and operational data to forecast microbial pollution risks, serving as an auxiliary early-warning tool rather than a direct microbial detection approach (Izah and Ogwu 2025). Its predictive accuracy is predominantly determined by the representativeness, completeness, and spatiotemporal coverage of historical training datasets. This review highlights that the selection of microbial detection methods should be tailored to specific monitoring goals, inherent water matrix properties, and practical resource conditions, rather than simply ranking the superiority of the aforementioned technical approaches. Table 4 summarizes the multidimensional trade-offs among these four core techniques.

### Image analysis and computer vision techniques

As a pivotal subdomain of AI, computer vision empowers automated parsing and quantitative analysis of image and video data, extracting actionable features to facilitate intelligent decision-making (Kalluri et al. 2025). In the domain of aquatic microbial detection, computer vision techniques are commonly integrated with microfluidic platforms to enable the automated processing and high-throughput analysis of microbial microscopic images, allowing for the precise characterization of microbial morphological traits (Zhou et al. 2023). Existing studies have demonstrated that computer vision-based AI approaches can substantially enhance the efficiency and precision of microbial image analysis, emerging as a core technical pillar for advancing automated aquatic microbial detection systems (Rauf et al. 2022). Table 1 shows selected examples of AI-enhanced microscopy techniques reported in the literature.

**Table 1:** AI enhanced microscopy technology for detecting waterborne pathogens

Technical approach	Core characteristics	Reported performance (with conditions)	Key limitations (verifiable)	References
Label-free 3D holographic tracking + deep learning	Continuous in-water monitoring; real-time data transmission	*Giardia lamblia* identification accuracy: 98%; 12-h advance warning for algal toxin-related behavioral anomalies (laboratory conditions; dataset size and validation protocol not fully reported)	Limited imaging resolution; positioning drift at depths > 30 m; unstable tracking in strong water currents	(El Mallahi et al. 2013; Göröcs et al. 2020)
Acoustic enrichment + label-free morphological identification	Biofouling-resistant surface design; device weight < 3 kg	*Legionella* detection in turbid water (specific turbidity value and validation protocol not reported)	Enrichment efficiency decreases by 40% at flow rates > 1 mL/min; inability to distinguish closely related bacterial species	(Screpis et al. 2024)
Digital holographic microscopy + CNN	Time-series imaging of viable bacteria	*Escherichia coli* detection limit: Approximately 1 CFU/L in low turbidity buffer solution (within 9 h)	Significantly reduced sensitivity in high-turbidity source water or raw wastewater; high risk of misclassification for closely related species in mixed microbial communities	(Wang et al. 2020)

The performance data comes from the main sources cited. Unless otherwise specified, indicators are obtained from standardized cultures in low turbidity matrices (sterile buffer or deionized water) under controlled laboratory conditions. Qualitative descriptors (such as “turbid water”) should be retained according to the report; The quantitative turbidity values in various studies are not consistent, which limits direct comparability. The listed technical methods are described in the cited sources

Current research in this domain has witnessed a gradual paradigm shift from conventional manual feature engineering to sophisticated end-to-end deep learning architectures (Ma et al. 2023). As a dominant backbone model in computer vision, convolutional neural networks (CNNs) are capable of autonomously learning discriminative latent features from microscopic images and have been increasingly deployed across diverse microscopic imaging platforms for aquatic microbial analysis (Zhang et al. 2023). Relevant empirical studies have demonstrated that, following pre-incubation of samples in selective culture media, CNN-integrated holographic imaging systems enable the detection of *Escherichia coli* at concentrations as low as 1 CFU/L within a 9-h detection workflow (Wang et al. 2020). Under standardized imaging conditions (fixed light source, 40 ×/100 × magnification microscope) and low turbidity laboratory conditions (clean glass slides, fixed staining, or simple culture medium smears) (dataset: approximately 15,000 microscopic images; validation: maintenance test set), Inception-V3 achieved a Top-1 accuracy of 82% for 10 fungi (Rahman et al. 2023); The average detection accuracy of YOLOv4 signal detection in clean water (< 5 NTU) is about 90–95% (Joshi et al. 2024); In laboratory environments, URV-SRS has achieved real-time imaging of nanoscale metabolic processes, providing a highly promising technological approach for cross-scale observation of microorganisms (Lin et al. 2025).

Nevertheless, diverse neural network architectures exhibit distinct applicability across practical application scenarios. Under standardized imaging protocols and low-turbidity laboratory conditions, classic CNN architectures exemplified by Inception-V3 achieve robust static microbial morphological classification, yielding classification accuracy ranging from 90 to 95% (Ma et al. 2023); however, for microorganisms exhibiting natural motion in liquid culture media, the recognition accuracy of these CNN models for motile bacterial cells declines by approximately 10%–15% (Ma et al. 2023). Equipped with real-time object-tracking modules, the YOLOv4 architecture elevates the recognition accuracy of motile microorganisms to 85%–90% in low- and medium-turbidity aquatic media, rendering it highly applicable to dynamic detection scenarios represented by flow cytometry analysis (Ma et al. 2023).

AI-powered microbial imaging has evolved along a clear technical pathway: from static microscopic morphology analysis, to dynamic target tracking, to high-throughput imaging flow cytometry, and finally to hyperspectral imaging. Among these, imaging flow cytometry serves as a key extension that enables continuous, real-time visual detection in flowing water systems.

Similarly, the integration of AI has driven substantial technological innovations in flow cytometry-based microbial detection (Zhang et al. 2024a, b, c). As documented in existing literature, portable imaging flow cytometry empowered by deep learning achieves rapid detection while preserving high resolution via high-speed holographic imaging and AI-assisted classification (Gӧrӧcs et al. 2018). For example, the integration of lensless holographic imaging flow cytometry and CNN architectures enables the high-speed analysis of 1000–3000 particles per second, achieving a classification accuracy of over 99% for typical waterborne pathogens such as Cryptosporidium and Giardia (Luo et al. 2021); under controlled laboratory conditions, this strategy supports rapid, label-free, and high-throughput analysis of natural aqueous samples, including lake and river water. For instance, the integration of virtual frozen fluorescence imaging flow cytometry (VIFFI) with deep learning algorithms achieves a classification accuracy of up to 98% for ten microbial species, including *Escherichia coli*, *Staphylococcus aureus*, *Pseudomonas aeruginosa*, and *Klebsiella pneumoniae*, as well as Cryptococcus (Mikami et al. 2020); this hybrid technique enables high-speed, high-resolution morphological profiling of fluorescently labeled microbes within microfluidic channels under liquid suspension conditions. In addition, the combination of flow cytometry and unsupervised machine learning (such as clustering analysis and self-organizing mapping) has been explored to detect microbial interference in drinking water microbial communities under different environmental conditions (such as changes in treatment processes and seasonal fluctuations) by continuously collecting high-dimensional cell count data (Props et al. 2018). As shown in Table 2. Overall, these advances provide tools for monitoring water source microorganisms, although their performance in routine field applications needs further validation.

**Table 2:** Deep learning imaging flow cytometry method for microbial detection in water sources

Technical approach	Core characteristics	Reported performance (with conditions)	Key limitations (verifiable)	References
Deep learning-assisted portable imaging flow cytometer	Label-free; high-throughput	Classification accuracy of *Cryptosporidium parvum* and *Giardia lamblia*: > 99% (approximately 12,000 images, fivefold cross validation, laboratory conditions, low turbidity water)	The performance may decrease in high turbidity or complex matrix water; Insufficient verification in routine on-site applications	(Göröcs et al. 2020; Luo et al. 2021)
Virtual frozen fluorescence imaging flow cytometry + deep learning	High-speed imaging in liquid suspension	Classification accuracy for 10 microbial species (including *E. coli*, *Staphylococcus aureus*): 98% (fluorescence-labeled, laboratory conditions)	Requires fluorescence labeling; high equipment cost; increased false positive rate in turbid water	(Mikami et al. 2020)
Unsupervised machine learning + flow cytometry	Continuous acquisition of high-dimensional cellular data	Detection of microbial community perturbations in drinking water (across different treatment processes and seasonal fluctuations, laboratory/small-scale field conditions)	Highly dependent on raw data quality; lacks standardized industry validation protocols; limited sensitivity to low-abundance microorganisms	(Props et al. 2018)

High precision is typically achieved under controlled conditions (e.g., specificity > 99% in buffer), with recorded degradation in turbid or complex matrices. Quantitative turbidity values are provided where reported in the source literature

In the field of hyperspectral imaging (HSI), the synergistic integration of HSI techniques and AI enables the label-free detection of microorganisms in laboratory research scenarios (Tao et al. 2023). For instance, low-cost hyperspectral imaging coupled with machine learning algorithms accomplishes high-accuracy classification of macroalgae under laboratory experimental conditions; The model is trained on a 4000-image dataset and validated via independent test subsets, further enabling remote sensing monitoring with a detection distance of up to 90 m (Allentoft-Larsen et al. 2025). Classification of Laboratory-Induced VBNC Microorganisms Based on Hyperspectral Microscopic Imaging and the EfficientNet-V2 Model (Papa et al. 2025). As shown in Table 3.

**Table 3:** Application of hyperspectral imaging combined with CNN for microbial detection in water sources

Technical approach	Core characteristics	Reported performance advantages (with conditions)	Key limitations (verifiable)	References
Microscopic hyperspectral imaging + CNN	400–1000 nm wavelength range; sub-micrometer spatial resolution	Single cell microbial identification; Differentiation of pigment types (laboratory conditions, approximately 4000 images in the dataset)	Requires laboratory-based sample pretreatment; slow scanning speed; extremely high equipment cost	(Q. Li et al. 2024a, b)
Hyperspectral microscopy + EfficientNet-V2 for VBNC microbial detection	Classification of laboratory-induced VBNC microorganisms	Classification of laboratory-induced VBNC microorganisms (dataset size and validation protocol not fully reported)	Detection performance for VBNC populations in natural aquatic environments is unconfirmed; lacks universal standard validation protocols	(Papa et al. 2025)

Remote sensing for direct microbial detection is still in the early stages of research. The advantages of the report are mainly demonstrated under controlled or ideal conditions. The prospect of remote sensing capability further highlights the non-contact and potential wide area monitoring advantages of this technology

In conclusion, compared with spectroscopic and genomic analytical approaches, image-enabled microbial detection exhibits prominent merits in on-site deployability and real-time monitoring capacity under feasible operational conditions. Nevertheless, vision-based detection techniques exhibit inferior biochemical resolution compared with Raman spectroscopy and metagenomic sequencing, struggling to differentiate phylogenetically closely related bacterial species.

### Discrepancies between controlled experimental validation and practical real-world scenarios

Despite remarkable advancements in relevant research, a persistent performance gap remains between laboratory-validated outcomes and practical field applicability. Such discrepancies are primarily manifested in three critical real-world constraints: high-turbidity aquatic environments, complex mixed microbial communities, and ubiquitous environmental noise interference.

The turbidity continues to decrease in detection performance. The portable holographic imaging platform can achieve a detection limit of approximately 1 CFU/L for *Escherichia coli* in sterile buffer solution; when applied to high-turbidity source water or raw wastewater, the detection sensitivity significantly decreases and the detection limit significantly increases compared to pure media (Wang et al. 2020). A three-dimensional microbial tracking system based on digital holographic microscopy (DHM) and deep learning has excellent microbial tracking and recognition capabilities in low-speed flow environments of pure media; However, when applied to natural high-turbidity river water, an increase in water flow rate will significantly increase the probability of microbial trajectory loss, significantly reducing the system’s microbial tracking accuracy (Matthews et al. 2024).

Mixed microbial population. At present, most AI detection models are trained only on a single bacterial pure culture sample. When applying a tracking model based on digital holographic microscopy combined with deep learning to mixed microbial systems, strains with similar genetic relationships are prone to identification confusion, and the detection effect of microbial groups will also significantly deteriorate (Matthews et al. 2024). On the one hand, the model training dataset has a single species type and insufficient generalization ability; On the other hand, the morphology and movement characteristics of closely related microorganisms are highly similar, which further interferes with the recognition results.

Environmental noise and site conditions. Temperature fluctuations, water flow disturbances, and biological pollution caused by long-term equipment operation can all increase optical background noise, distort imaging features, and reduce microbial identification accuracy (Calero Preciado et al. 2021; Xu et al. 2025). At present, most relevant studies only conduct controllable experiments under a single interference condition, and there is a lack of comprehensive verification for multiple on-site interference superposition conditions.

In summary, existing studies seldom perform thorough analyses of performance deterioration, identification bias and insufficient experimental reproducibility. These limitations may consequently result in an overestimation of the practical applicability of the corresponding detection methods.

All reported performance degradations are based on laboratory simulations; direct field quantification in real aquatic environments remains rarely reported in current literature.

### AI-enhanced spectroscopic analytical techniques

Traditional spectroscopic detection techniques profile unique chemical fingerprints based on light-matter interactions (Shi et al. 2026). Nevertheless, their practical performance is substantially constrained by background interference, spectral signal overlap, and dependence on manual spectral interpretation, thereby compromising detection accuracy within complex aquatic matrices. AI mitigates the above constraints through automated spectral denoising, feature extraction and pattern recognition, leading to enhanced detection sensitivity and specificity (Huang et al. 2022; Srivastava et al. 2024). This synergistic combination has promoted the evolution from offline manual spectral interpretation to real-time data-driven detection.

In Raman spectroscopy, AI exhibits powerful performance under controlled laboratory conditions. For instance, deep learning models coupled with single-cell Raman spectroscopy enable high-precision bacterial classification under pure culture laboratory conditions, achieving a maximum species-level identification accuracy of 96% (Li et al. 2025). However, the transfer of these models to complex environmental samples leads to an apparent decline in overall recognition performance. Such performance deterioration in mixed microbial communities represents a critical bottleneck for the practical analysis of natural water samples with diverse microbial compositions. Laser tweezers Raman spectroscopy combined with CNN can achieve 100% accuracy in classifying five spore species of Bacillus subtilis from pure culture sources (Du et al. 2023). This study has only been validated in a laboratory pure culture system and has not yet been applied to the detection of spore samples in environmental water samples and natural conditions.

The integration of AI also optimizes surface-enhanced Raman spectroscopy (SERS), enabling high-accuracy microbial detection. For instance, SERS utilizing gold nanorods combined with machine learning attains an identification accuracy above 87% when differentiating four bacterial species in filtered and sterilized wastewater (Herndon et al. 2025). Meanwhile, SERS with multifunctionally modified substrates coupled with a one-dimensional convolutional neural network (1D-CNN) delivers a classification accuracy of 99% across 17 bacterial species under standardized culture conditions (Liu et al. 2025). Notably, SERS substrates based on silver nanorods combined with the Raman network model enable the accurate classification and identification of trace bacterial endotoxins in buffer solutions (Yang et al. 2022). Variations in the matrix environment can interfere with Raman signal responses. Despite advances in SERS-based detection techniques, existing research has mainly been validated in laboratory environments using simple, low-interference matrices. In addition, most published studies have failed to provide detailed explanations of dataset features, model validation protocols, and water-based matrix parameters, seriously hindering the generalizability evaluation of the model in practical field scenarios.

Comparative analyses across gradients of microbial community complexity highlight the necessity of standardized environmental condition reporting for method validation. Support Vector Machine (SVM) algorithms can yield high classification accuracy for single bacterial strains in wastewater matrices; nevertheless, the coexistence of multiple bacterial species in mixed systems degrades model recognition performance relative to pure-strain samples (Herndon et al. 2025). Similarly, the integration of one-dimensional convolutional neural networks (1D-CNN) with multi-channel feature fusion strategies exhibits outstanding classification capacity for pure-cultured bacterial specimens and maintains superior overall identification accuracy (Liu et al. 2025). Importantly, such promising analytical outcomes are exclusively derived from well-controlled laboratory-prepared samples and cannot be directly generalized to untreated raw environmental water samples.

In conclusion, AI-enhanced spectroscopic techniques have validated the technical feasibility of microbial detection under controlled laboratory environments. Nevertheless, their practical applicability in complex aquatic matrices, including turbid surface water and wastewater, remains to be further verified. Major bottlenecks primarily involve unstable signal reproducibility in varied matrix systems, interference effects induced by coexisting substances, and the absence of unified reporting criteria for dataset attributes, model validation paradigms, and environmental parameters in most preliminary investigations.

### Deficient sample representativeness, insufficient technical reproducibility, and inadequate anti-interference validation for complex aquatic matrices

Due to the lack of sample representativeness, repeatability, and anti-interference validation, the actual performance estimation of this technology is overestimated, which hinders its promotion:

Existing samples exhibit inadequate representativeness, as the superior detection performance of relevant techniques is predominantly achieved based on standardized culture or artificially configured samples (Srivastava et al. 2024). Such sample conditions fail to conform to the actual stress status of bacteria and the intricate characteristics of natural aquatic microbial communities. Practical detection accuracy is likely to decline, and the adaptability of these analytical approaches remains uncertain. Furthermore, the resultant deviations have not been fully quantified.

Poor technical repeatability. SERS exhibits unsatisfactory reproducibility, as its analytical performance is highly susceptible to nanostructured substrate fabrication (Sloan-Dennison et al. 2024). Currently, Raman spectroscopic detection lacks universal parameter optimization schemes and standardized experimental guidelines, making cross-study result comparison difficult. These challenges constitute a major bottleneck for the practical deployment and popularization of the technique.

Insufficient anti-interference verification. SERS detection achieves favorable performance in clean, low-interference systems including deionized water and buffer solutions. Natural organic matter such as humic substances and proteins present in aquatic environments can alter the microdistribution of target analytes at the nanosubstrate interface (Yang et al. 2023). This induces signal interference and baseline drift, lowering detection specificity and raising the probability of false-positive results. The evident discrepancy in quantitative performance highlights that specificity assessment under realistic interference conditions is an urgent research priority.

Despite the capability of rapid preliminary screening, imaging and spectroscopic techniques fail to identify uncharacterized microbial taxa or trace low-abundance pathogens and functional genes. In contrast, genomic and metagenomic sequencing enables direct acquisition of microbial genetic profiles, while AI facilitates the high-throughput, high-precision interpretation of these intricate omics datasets.

### AI-driven analytical strategies for genomic and metagenomic data mining

High-throughput sequencing technology can directly obtain microbial genetic information from environmental water samples without the need for microbial cultivation (Venter et al. 2004). The integration of AI algorithms, particularly deep learning, with high-throughput sequencing enables efficient and systematic metagenomic data analysis. This synergistic strategy facilitates the precise identification of aquatic pathogenic microorganisms, antibiotic resistance genes, and functionally distinct microbial communities in water environments (Mahajna et al. 2022). Machine learning approaches also enable the efficient mining of microbial interaction networks and rare microbial population signals, providing robust theoretical and technical support for practical applications including waterborne disease early warning and pollution source tracing (Mahajna et al. 2022).

The functional roles of AI differ substantially across diverse genomic analytical approaches. This section categorizes four major types of omics data based on biological data characteristics, AI task orientations, and environmental monitoring objectives.

#### Shotgun metagenomics (Otten et al. 2016)

Biological data type: whole-genome DNA sequences derived from all microbial taxa within environmental samples. AI tasks: sequence assembly and merging, taxonomic classification, and functional annotation of microbial genomic data. Monitoring purposes: pathogenic microbial identification, spatiotemporal surveillance of antibiotic resistance genes, and environmental pollution source attribution. The application of metagenomic approaches in aquatic microbial ecology research facilitates the exploration of novel uncultured microorganisms and their unique metabolic pathways, while advancing the establishment and improvement of systematic aquatic and marine metagenomic databases (Venter et al. 2004). Leveraging taxonomic profiles and functional gene abundance datasets, machine-learning algorithms enable the automatic extraction of microbial biomarkers, accurate prediction of host phenotypic traits, and in-depth functional profiling of microbial communities (Sharma et al. 2025).

The integration of targeted amplicon sequencing, represented by 16S rRNA gene sequencing, with machine learning algorithms including random forest and neural networks enables effective structural analysis of microbial communities (Rekadwad et al. 2024). Furthermore, deep learning frameworks such as Virtifier, PathoScope 2.0, and CNNs exhibit high sensitivity in the identification and detection of diverse pathogenic microorganisms (Blaustein et al. 2025; Roy et al. 2024). In core bioinformatics workflows encompassing sequence binning, dimensionality reduction analysis, and functional annotation, AI tools have significantly enhanced the efficiency and precision of omics data analysis. Accordingly, AI-driven metagenomic analysis has emerged as a pivotal technical pillar for ecological security monitoring of aquatic environments.

#### Transcriptomics (McCarren et al. 2010)

Biological data type: Transcriptomic RNA sequences corresponding to actively expressed functional genes. AI tasks: Taxonomic classification of metabolically active microorganisms and targeted identification of functional genes. Monitoring objective: to identify the VBNC population and active functional genes. The integration of transcriptomic profiling with SVM algorithms enables the precise identification of metabolically active microorganisms and their functional genes (Shi et al. 2009).

#### Single-cell genomics (Baek et al. 2025)

Biological data type: Genomic sequences derived from pure-cultured and isolated microbial strains. AI tasks: Microbial cell clustering and identification of rare taxonomic groups. Monitoring objectives: Detection of rare microbial taxa and waterborne parasitic pathogens. The integration of single-cell genomics with AI approaches offers a high-precision analytical solution for the detection of low-abundance and rare microbial taxa (Dam et al. 2020).

In contrast to imaging and spectroscopic techniques, metagenomic sequencing delivers superior taxonomic classification and functional resolution, which cannot be accomplished by morphological observation or conventional molecular fingerprinting alone. Nevertheless, its high operational costs, prolonged sequencing turnaround time, and heavy dependence on curated reference databases constrain its applicability in routine rapid screening, restricting it primarily to targeted monitoring and pollutant source tracking analyses.

### Shortage of data resources, limited algorithm scenarios, and insufficient support for result validation and interpretation

Data dependency and resource constraints represent prominent technical bottlenecks. Several state-of-the-art analytical approaches require massive sequencing datasets, which greatly restricts their practical deployment in resource-limited and remote regions. The coverage of freshwater and wastewater samples in existing metagenomic repositories remains limited, and datasets pertaining to rare microbial taxa are notably deficient. Accordingly, the accuracy of functional annotation tends to decline for non-marine aquatic samples. The magnitude of such performance degradation varies with sample categories and the completeness of the adopted reference databases.

Limited adaptability to specific analytical technology scenarios. Targeted sequencing and related methods are incompetent for discovering novel genes. Single-cell analytical techniques are not applicable to large-scale monitoring. Moreover, rare microbial specimens characterized under laboratory conditions may diverge from their in-situ states in natural aquatic matrices.

Insufficient interpretation and validation of results. Current AI-assisted metagenomic analytical approaches suffer from insufficient interpretability regarding their internal decision mechanisms, and relevant studies generally lack cross-validation based on independent datasets. In the detection of low-abundance antibiotic resistance genes from wastewater metagenomic data, pipelines with rigorous multi-source validation protocols exhibit substantially lower false discovery rates than those relying solely on single-dataset training and testing partitioning. Such inherent limitations underscore the urgent need for standardized and unified verification frameworks for AI-driven metagenomic analysis.

Imaging, spectroscopic, and genomic techniques characterize the real-time status of aquatic environments. By integrating historical and real-time environmental, operational and molecular datasets, predictive modeling further extends this capacity to forecast potential pollution risks.

### Microbial contamination prediction model

A clear distinction should be made between pollution risk forecasting and direct microbial detection, since the two research objectives are correlated yet fundamentally distinct (Holcomb and Stewart 2020). Direct analytical approaches including imaging, spectroscopy and genomics enable the identification of target pathogens within samples at specific time points. In comparison, predictive modeling bypasses direct microbial quantification. Instead, it integrates environmental parameters, operational conditions, and retrospective pollution records to forecast the occurrence probability and potential magnitude of subsequent pollution events (Walsh et al. 2024).

When furnished with sufficient high-quality historical datasets and applied within the defined training boundaries, AI algorithms are able to identify non-linear patterns undetectable by traditional statistical techniques (Yu et al. 2024). These models enable real-time or near-real-time pollution alerting and trend forecasting. Nevertheless, the reliability and generalizability of their outputs are environment-dependent, and cross-environment application requires rigorous prior validation.

From an algorithmic perspective, machine learning approaches including SVM, random forest (RF), gradient boosting classifier (GBC), and artificial neural network (ANN) have been extensively applied to decipher complex interactions within high-dimensional biological features (Ghannam and Techtmann 2021; Stocker et al. 2021). For instance, machine learning facilitates the tracing of primary water pollution sources, enabling source identification and early warning under field conditions (Wu et al. 2020). In a practical case of urban water distribution networks, the Gated Graph Neural Network (GGNN) can pinpoint suspected pollution nodes using 2-h monitoring datasets, thereby significantly narrowing the scope of emergency investigations (Li et al. 2024a, b).

Nevertheless, the accuracy and reliability of such predictive outputs are predominantly derived from investigations conducted under controlled and site-specific constrained conditions. Consequently, these findings cannot be generalized across diverse geographical regions, varied aquatic substrates, and distinct seasonal scenarios. As elaborated in the subsequent section, predictive models present notable limitations regarding the spatiotemporal representativeness of datasets, model generalizability and interpretability.

In summary, predictive modeling is fundamentally distinct from the three detection-oriented analytical approaches, as it weakens the need for direct microbial detection. Conversely, predictive modeling provides an auxiliary warning dimension by inferring pollution risks from multi-source environmental and operational profiles. Its practical applicability is predominantly constrained by the quality, spatiotemporal comprehensiveness, and representativeness of training data.

### Insufficient spatiotemporal representativeness of data, weak model generalization ability, and lack of interpretability

Deficiencies in data quality and representativeness undermine the reliability of predictive outputs. The development of predictive approaches depends on retrospective monitoring datasets. Limited spatiotemporal coverage, incomplete pollution archives and deficient data on low-concentration contaminants collectively elevate prediction errors for mild and abrupt pollution episodes.

These predictive approaches exhibit inadequate generalizability and robustness. Models trained on single regional datasets tend to suffer from degraded predictive accuracy and elevated prediction errors when deployed in geographically distinct regions. Furthermore, these models are susceptible to anomalous data. A small fraction of outliers induced by extreme precipitation, device malfunctions and other factors can impair the reliability of predictive results. During seasonal transitions and drastic variations in environmental conditions, water quality parameters that fall outside the typical range of training data will further aggravate the decline in model performance. Current studies have failed to comprehensively resolve the inherent robustness limitations of predictive models, including spatial transfer capability, anti-interference performance against anomalous disturbances, and seasonal adaptability.

Insufficient interpretability of decision-making logic. Certain predictive approaches suffer from low interpretability in their decision-making mechanisms, thereby undermining the credibility of relevant public health decision-making. Furthermore, inadequate uncertainty quantification and insufficient traceability analysis further weaken the practical applicability of these models in real-world scenarios.

All the aforementioned applications are built upon core AI algorithms. The subsequent section systematically elaborates on deep learning architectures, which excel at analyzing complex datasets including images, spectra and biological sequences, and have substantially boosted analytical performance.

**Table 4:** Context-dependent trade-offs in AI-enhanced detection of waterborne microorganisms

Comparison dimension	Imaging	Spectroscopy (Raman/SERS)	Genomics/metagenomics	Predictive modeling
Detection principle	Morphological features	Molecular vibrational fingerprints	DNA/RNA sequences	Environmental data correlation
Relative sensitivity	Medium	High (under optimized conditions)	Extremely high (single-copy level)	No direct detection capability
Relative specificity	Low (difficult to distinguish closely related species)	Medium–high (database-dependent)	High (strain-level resolution)	Not applicable
Typical detection time	Minutes to hours	Minutes to hours	Hours to days	Real-time/near real-time
Field deployment feasibility	Relatively high (portable devices commercially available)	Medium (dependent on instrument stability)	Low (laboratory-only)	High (only requires sensor input)
VBNC detection capability	Undetectable	Can measure metabolic activity (pending validation)	Limited (requires RNA sequencing)	Not applicable
Relative cost	Low–medium	Medium	High	Low (data-dependent)
Data requirement	10^4^ labeled images	Hundreds to thousands of spectra	10 Gb of sequencing data	High-quality historical monitoring data
Typical applicable scenarios	Rapid screening of low-turbidity water	Low-pollution water, closely related species identification	Source tracking, antibiotic resistance gene surveillance	Early warning, trend prediction
Field validation level	Limited field validation available	Mostly laboratory-based	Almost exclusively laboratory-based	Highly dependent on data representativeness

All performance metrics presented in this table are obtained under ideal experimental conditions, namely low turbidity, standardized cultivation and adequate training datasets

Table 4 summarizes the contextual trade-offs among the four detection approaches under ideal laboratory settings. Table 5 further presents a quantitative comparison of core deployment indicators for representative systems documented in existing literature, covering economic cost, analytical throughput, energy consumption, and device portability.

**Table 5:** This quantitative comparison evaluates all methods in terms of cost, analytical throughput, energy consumption and portability

Method category	Single analysis cost	Throughput	Power consumption	Portability (weight)
Imaging (flow cytometry)	$0.60–720	10,000 events/sec; 1 L/5 min	< 60–3,000 W	Benchtop (3–25 kg)
Imaging (holography)	< $0.60	Real-time continuous	Solar-compatible	Portable (< 3 kg)
Spectroscopy (hyperspectral)	$200 (laboratory)	Slow (minutes to hours)	500–1000 W	Laboratory-only
Genomics (metagenomics)	$250–600 per sample	Hours to days	High	Laboratory-only
Predictive modeling	< $1 (computation only)	Real-time/near real-time	< 50 W	Sensor-dependent

Some of the data in this table, such as flux, detection time, and portable weight, are sourced from Tables 1, 2 and 3 and references cited in Sect. 2 of the main text; Cost and energy consumption data are mostly reasonable estimates made by the author based on equipment types, reagent consumption, and industry pricing. They vary by region, sample size, and procurement channels and are for reference only. Samples corresponding to the cost of metagenomics; The cost of predictive modeling assumes the existing sensor infrastructure

### Deep learning architecture

Deep learning, a pivotal subdomain of machine learning, leverages multilayer neural network architectures to process and model complex high-dimensional datasets (LeCun et al. 2015). Previous sections have elaborated on deep learning implementations across diverse detection modalities, including imaging analysis, spectroscopic detection, and genomic data processing. This part highlights cross-domain considerations for deep learning methodologies, irrespective of data categories.

Nevertheless, the implementation of deep learning in aquatic microbial detection inevitably suffers from several inherent limitations. First and foremost, deep learning models generally rely on large-scale annotated datasets to attain the high accuracy levels documented in existing comparative investigations (Høye et al. 2022; Tan et al. 2025). Under limited training data scenarios (100–400 annotated images per species), existing studies have demonstrated that the classification accuracy for morphologically similar planktonic species can decline by 18%–25% relative to models trained with sufficient datasets (Maracani et al. 2023). Secondly, intensive computational demands hinder the on-site deployment of deep learning models. Parameter-heavy architectures with tens of millions of parameters, such as ResNet-152, occupy substantial GPU memory resources. When deployed on conventional GPU-free laptops, the model inference latency can increase from minutes to hours, severely restricting its applicability in rapid response scenarios (Pu et al. 2023). Thirdly, deep learning models are inherently constrained by low interpretability, which remains a critical bottleneck for practical applications. Although post-hoc interpretation methods, including saliency mapping (e.g., Grad-CAM) and feature attribution algorithms (e.g., SHAP), enable visual and quantitative explanation of model decisions, these techniques introduce additional computational overhead, and their explanatory fidelity requires task-specific validation (Chabrier et al. 2024; Skliarov et al. 2025). Current studies predominantly prioritize predictive performance while overlooking several key drawbacks of deep models, including high computational and data dependence, insufficient interpretability, and potential privacy risks. Such privacy concerns primarily stem from analytical biases induced by data anonymization operations and imbalanced training datasets.

Despite the aforementioned challenges, deep learning serves as a robust analytical tool given adequate data and computational resources, particularly for tasks leveraging automated feature extraction. Transfer learning and self-supervised learning represent promising emerging strategies to mitigate reliance on labeled data. Nevertheless, their applications in aquatic microbial detection remain at an early developmental stage. Nevertheless, traditional machine learning still plays an irreplaceable role. Featuring light computation, favorable interpretability and strong adaptability to small datasets, it is widely adopted for routine monitoring and deployment in resource-limited scenarios (Pluym et al. 2024).

### Classic machine learning

Classical machine learning algorithms, such as SVM, RF, GBC, and KNN, have been widely applied to aquatic microbial detection (Weller et al. 2021). These approaches are particularly suitable for scenarios characterized by limited training samples, high demand for model interpretability, and constrained computational resources. As mentioned in previous sections, these classical algorithms have been extensively adopted for pollution prediction modeling and metagenomic feature selection. This section systematically explores the cross-domain and cross-modal constraints that restrict the practical performance of classical machine learning algorithms.

Several inherent limitations persist when machine learning techniques are implemented for detection tasks. First, high-dimensional data combined with insufficient samples tends to induce overfitting in SVM. Meanwhile, KNN suffers from elevated computational complexity when processing large datasets, making it ill-suited for high-throughput screening workflows. Secondly, class imbalance poses persistent challenges to model performance. For scenarios where target microbial taxa exhibit low relative abundance within microbial communities, existing studies have verified that RF and GBC algorithms suffer from degraded recall performance (McElhinney et al. 2022). This issue arises because such models tend to misclassify rare microbial cells into dominant species with analogous feature embeddings. Thirdly, feature engineering still occupies a critical position in conventional machine learning pipelines. Classical machine learning algorithms lack the capability of automated feature learning, and their predictive performance is heavily contingent on the quality of manually engineered input features (Lamrini et al. 2024). Feature pipelines optimized for a specific water matrix, such as drinking water, often fail to generalize effectively to other matrices like wastewater. Correspondingly, model accuracy tends to decline in cross-matrix validation, leading to poor cross-scenario transferability.

Given the aforementioned trade-offs, classical algorithms are best suited for scenarios with moderate data scale, well-defined features and high demands for model interpretability. Hybrid frameworks adopt classical algorithms for preliminary screening and leverage deep learning for subsequent targeted verification. This strategy shows great theoretical potential, yet its on-site validation remains in an early phase.

### Towards the development of an integrated detection pipeline

**Fig. 2:**
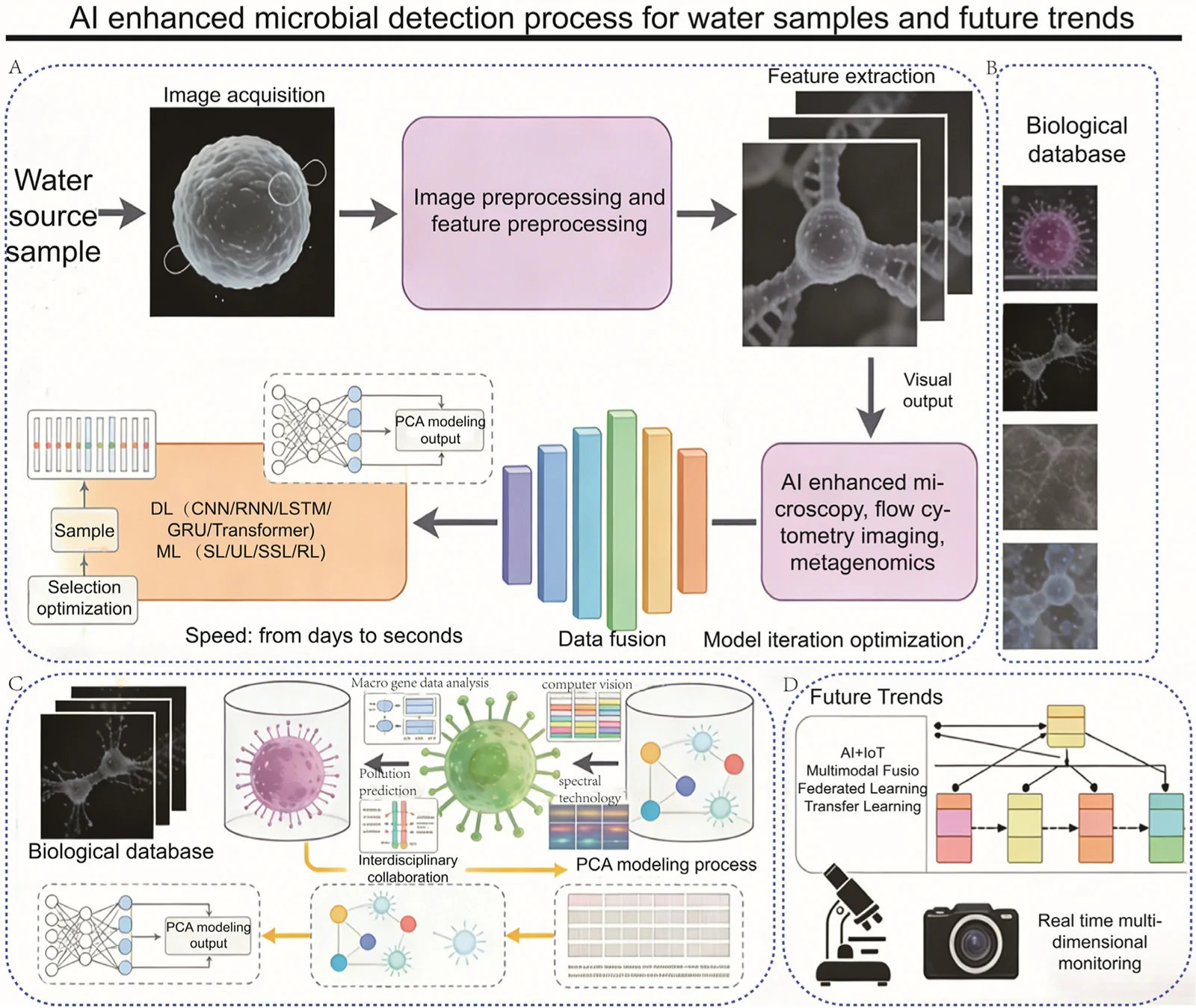
Overall framework for integrated water quality detection pipelines

While previous sections have elaborated on imaging, spectroscopy, genomics, and predictive modeling as independent technological methodologies, practical water quality monitoring scenarios seldom depend on a single analytical technique. Instead, these approaches exhibit inherent complementarity. Their systematic integration enables the construction of multi-stage detection pipelines that comprehensively balance detection efficiency, analytical resolution, and economic cost under diverse operational constraints (Fig. 2).

The architectural configuration of this integrated conceptual pipeline is detailed as follows:

- Screening stage (imaging-based): For low-turbidity water environments and field scenarios requiring rapid response, AI-augmented imaging techniques, including portable holographic imaging and flow cytometric systems, can serve as primary preliminary screening tools. Despite relatively limited taxonomic resolution, such systems enable the rapid identification of candidate microbial targets within minutes to hours.
- Confirmation stage (spectroscopy and genomics-based): When imaging screening indicates potential pathogen presence or necessitates species-level identification, including the detection of VBNC states and antibiotic resistance genes, equivalent or subsampled water aliquots can be further analyzed via Raman/SERS spectroscopy and metagenomic sequencing. These analytical modalities provide high-precision molecular and genetic fingerprinting information, yet they entail prolonged turnaround durations ranging from hours to days and elevated economic costs.
- Prediction stage (predictive modeling-based): Synchronous with microbial detection procedures, AI models trained on historical environmental and operational datasets enable the early prediction of water pollution risks, such as forecasting Escherichia coli outbreaks 24 to 48 h in advance. While such predictive outputs cannot substitute direct analytical detection, they facilitate targeted sampling strategies and deliver proactive risk early warnings for water quality supervision.

It is worth emphasizing that full automation of these integrated workflows has not been realized on most current platforms. Inter-stage transitions largely depend on manual sample handling, data format conversion and standalone model execution (Burnet et al. 2025). For example, positive signals acquired from imaging modules fail to automatically trigger subsequent genomic validation in practical applications. Moreover, predictive models run on separate data pipelines including sensor time-series datasets, and real-time integration with detection outputs remains unimplemented. Accordingly, while a unified analytical pipeline is theoretically viable and highly promising, its practical deployment remains an active research area. Major obstacles include incompatible data schemas, the absence of standardized instrument interfaces, and insufficient field validation assessing the end-to-end performance across all three analytical stages. Resolving these integration bottlenecks is as critical as optimizing individual AI models.

## Overcoming the application challenges of AI in microbial detection in water sources

The preceding section systematically reviewed the technological advances of imaging, spectroscopy, genomics, and predictive algorithms, while pinpointing prevalent limitations regarding data quality, model robustness, interpretability, and field adaptability. These bottlenecks are not confined to individual techniques but represent cross-domain barriers hindering the large-scale practical deployment of water monitoring systems.

Despite the great potential of AI for aquatic microbial detection, its large-scale field deployment remains hampered by multiple interrelated core challenges. A fundamental bottleneck is the scarcity of high-quality datasets, particularly those with precise annotations, ecological representativeness and unified standardization. This issue is further compounded by practical constraints, including sparse monitoring coverage, substantial background noise in raw readings, and incompatible data formats across distinct research and monitoring programs. Collectively, these factors impede the training of robust models and degrade their generalization performance across diverse aquatic environments.

Tied closely to data-related constraints are the deficiencies in model design and operational performance. Despite the superior predictive capacity of sophisticated models such as deep neural networks, they are widely regarded as “black-box” systems owing to their low interpretability (Gao et al. 2025). Though explainable AI (XAI) techniques have been developed to enhance decision transparency, their practical deployment in aquatic microbial detection remains immature (Ligda et al. 2024). Addressing this issue necessitates the development of task-specific XAI strategies tailored for water quality monitoring. Two representative approaches are widely applicable: (1) Saliency mapping techniques (e.g., Grad-CAM), which visualize critical image regions contributing to pathogen classification. This paradigm enables microbiologists to validate that model inferences rely on biological characteristics such as cellular morphology rather than irrelevant background artifacts; and (ii) feature attribution algorithms (e.g., SHAP) quantify the marginal contributions of environmental variables, including turbidity, precipitation, and temperature, to pollution prediction models, thereby enabling targeted and interpretable regulatory interventions. Nevertheless, each of these methods introduces extra computational overhead, and their explanatory fidelity demands task-specific validation using water environment-related datasets. The absence of XAI embedding can impair public health decision-making. Uninterpretable false positives may result in redundant wastewater management alerts, while unaccounted false negatives will postpone epidemic intervention, erode regulatory credibility and impede practical field deployment. An inherent trade-off persists among model interpretability, computational efficiency, and predictive accuracy, creating substantial barriers to field deployment that demand simultaneous optimization of predictive performance and interpretability.

This issue is further complicated by the limited robustness of most existing models against dynamic real-world conditions. It thus underscores the urgent requirement to improve cross-scenario applicability via advanced techniques including transfer learning and causal reasoning. Accordingly, insufficient reproducibility stems from three primary factors. First, the absence of standardized datasets: most reported high accuracy is achieved using water samples with low turbidity (< 5 NTU) and over 10^4^ images per category, while model performance degrades when applied to alternative datasets or highly turbid water (> 50 NTU); (2) Discrepancies in experimental protocols and variable flow rates ranging from 0.5 to 1.5 mL/min can degrade tracking accuracy, yet such critical experimental parameters are frequently underreported in existing studies; (3) Inconsistent validation strategies: fewer than 10% of existing studies adopt independent external validation, which generally yields lower predictive accuracy than internal validation approaches.

Beyond technical limitations, insufficient interdisciplinary integration and defective governance mechanisms further impede the advancement of relevant research and applications. The development and field deployment of robust AI-driven detection systems necessitate in-depth interdisciplinary collaboration across multiple disciplines: microbiology for elucidating the inherent characteristics of target microbial communities, environmental science for interpreting complex aquatic matrix interference effects, and computer science for optimizing model architectures. Collectively, these requirements impose stringent demands on interdisciplinary synergy and cross-domain knowledge fusion.

Meanwhile, ethical norms and data governance also deserve close attention. Formulating standardized data specifications, advancing responsible data sharing, and implementing privacy-preserving techniques including federated learning and blockchain are essential to safeguard data security. Equally vital is the formulation of ethical frameworks to mitigate algorithmic bias. These components collectively underpin responsible AI implementation in microbiological detection. Accordingly, resolving these interconnected challenges is a prerequisite for translating lab-scale AI research into dependable real-world monitoring systems.

To systematically tackle the aforementioned limitations, this study proposes a three-tier integrated framework as a prospective developmental roadmap:

- Data layer: Future research should prioritize the construction of a unified multi-matrix database covering freshwater, seawater, and groundwater scenarios, alongside the adoption of standardized annotation protocols to enhance overall data consistency and generalizability. To alleviate sample scarcity for rare taxonomic taxa, synthetic datasets exemplified by GAN-generated data can act as effective supplementary resources, though their practical applicability requires further empirical validation.
- Algorithm layer: Future investigations should explore lightweight multimodal architectures that fuse multi-source data to comprehensively characterize microbial phenotypic features. Knowledge extraction techniques offer a viable paradigm for compressing sophisticated high-performance models into efficient lightweight variants, thereby reconciling predictive accuracy with computational feasibility to facilitate on-site practical deployment.
- Application layer: The research community is recommended to establish standardized validation criteria for AI models, which encompass cross-matrix testing across diverse aqueous media (e.g., drinking water, surface water, and wastewater) and evaluation based on sufficiently large independent sample datasets to quantify model reliability under real-world operational conditions.

This integrated framework delivers a holistic roadmap for addressing the technical and systemic barriers that currently decouple lab-scale AI research from large-scale microbial monitoring applications in aquatic environments. Despite the universality of these core challenges, their severity and adaptive optimization requirements vary substantially across distinct water body types (i.e., drinking water, surface water, and wastewater), driven by heterogeneous water quality matrices, differentiated risk thresholds, and varied pathogen transmission pathways.

### Challenges brought by water types

**Fig. 3:**
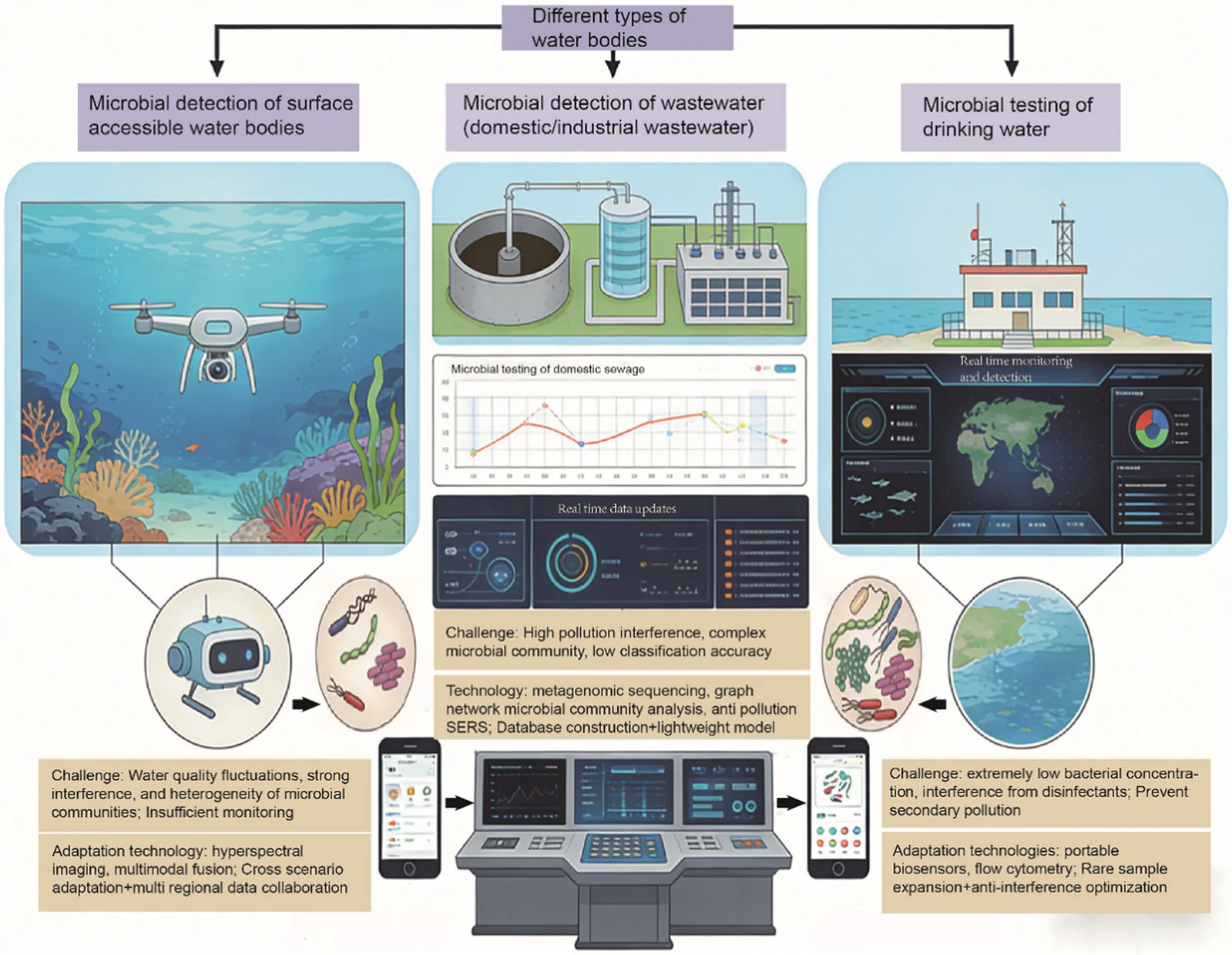
Elaborates on AI-aided detection technologies and their core technical bottlenecks, visually illustrating the differentiated adaptive mechanisms for microbial safety monitoring across diverse aquatic matrices

The functional attributes, transmission routes, and water quality profiles of diverse water bodies dictate the primary technical bottlenecks for the adaptive deployment of microbial detection techniques, as illustrated in Fig. 3. The detailed elaborations are presented below.

#### Drinking water

- Transmission route: from water sources through regulatory governance and pipeline distribution networks to end-user access points
- Primary risks: consist of low pathogen abundance, VBNC phenotypes, and pipeline-derived secondary contamination
- Adaptation challenges: involve extremely low target microbial concentrations, prominent interference induced by residual disinfectants, insufficient rare-sample datasets, and the necessity of anti-interference optimization

#### Surface water

- Transmission route: Human exposure via dermal contact and ingestion
- Primary risks: encompass drastic environmental fluctuations, intense background matrix interference, and pervasive spatiotemporal heterogeneity
- Adaptation challenges: stem from sparse monitoring sites that result in limited data representativeness

#### Wastewater

- Transmission routes: sequential propagation from power generation and wastewater treatment to discharge or reuse, ultimately triggering secondary environmental exposure
- Primary risks: include high pollutant loads, complex microbial consortia, prevalent antibiotic resistance genes (ARGs), and severe spectral signal interference
- Adaptation challenge: Degraded classification performance in complex mixed microbial consortia

### Regulatory considerations

Beyond the technical bottlenecks inherent to specific aquatic matrices, regulatory deficiencies constitute another cross-disciplinary barrier hindering the practical deployment of relevant techniques. This review fails to comprehensively elaborate on the regulatory framework governing practical field deployments. Accordingly, this section systematically summarizes the current guidelines pertaining to AI-based aquatic microbial detection technologies.

### AI based microbial detection

Currently, no unified international regulatory criteria are specifically designated for AI-based aquatic pathogen detection (Sisay et al. 2024). Nevertheless, several applicable and emerging normative documents are available as follows:

Regulation (EU) 2017/746 on In Vitro Diagnostic Medical Devices (IVDR) stipulates that AI-enabled detection systems deployed for public health surveillance purposes may be categorized as in vitro diagnostic medical devices, mandating standardized performance assessment and continuous post-market surveillance.

#### Research gap

Currently, no standardized regulatory pathway authorizes AI-derived detection outcomes as standalone compliance evidence for water quality criteria (e.g., drinking water standards). Accordingly, AI-enabled analytical tools remain auxiliary early-warning instruments that mandate confirmatory laboratory testing for authoritative validation.

### Water safety monitoring

Current water safety governance frameworks lack explicit incorporation of AI-driven analytical and monitoring paradigms.

The World Health Organization (WHO) Water Safety Plans (WSPs) stipulate that AI techniques can function as valid auxiliary analytical tools upon rigorous validation against standard reference methods; nevertheless, unified acceptance criteria for AI-generated alert signals remain absent.

The ISO 5667 series lacks standardized protocols governing the acquisition of AI training datasets, including requirements for spatiotemporal representativeness and unified annotation specifications.

#### EU regulation

2020/741 on water reuse defines conventional monitoring frequencies but fails to address the substitution of traditional detection methodologies with AI techniques.

#### Research gap

Most regulatory jurisdictions have not established unified criteria for the equivalence recognition and supplementary application of AI monitoring technologies.

### Data governance and privacy

AI-enabled monitoring has introduced a series of unaddressed governance challenges within the prevailing water regulatory systems.

Data ownership and data sharing: mechanisms remain undefined, with a lack of standardized protocols supporting multi-institutional collaborative AI training based on water quality datasets.

#### Privacy risks

High-resolution spatiotemporal monitoring data may indirectly infer household-level behavioral characteristics. Once such datasets are geolocally traceable and individually identifiable, they fall within the scope of the storage limitation requirements (Article 5) and data security obligations (Article 32) stipulated in the General Data Protection Regulation (GDPR). Similar regulatory provisions are also specified in China’s Personal Information Protection Law (PIPL).

#### Algorithm bias and transparency risks

In accordance with Article 6 of the EU AI Act, AI systems deployed for critical water infrastructure management are categorized as high-risk AI systems, mandating systematic qualification assessment and continuous human oversight. On-site evaluation reports determine the applicable scenarios of AI systems, including regulatory compliance verification, internal early warning, and academic research applications.

It is essential to quantitatively characterize the performance of complex aquatic matrices and elaborate on the inherent insufficient representativeness of existing datasets. Timely collaboration with competent regulatory authorities is urgently required to establish a standardized technical verification framework.

## Future perspectives and emerging research trends

The aforementioned challenges, including data sparsity, limited model generalizability, inferior model interpretability, and inadequate on-site validation, substantially shape the future development trajectory of relevant technologies. Each emerging technological trend targets a specific technical bottleneck, advancing AI-driven microbial detection toward real-time, intelligent, and sustainable aquatic monitoring paradigms.

**Fig. 4:**
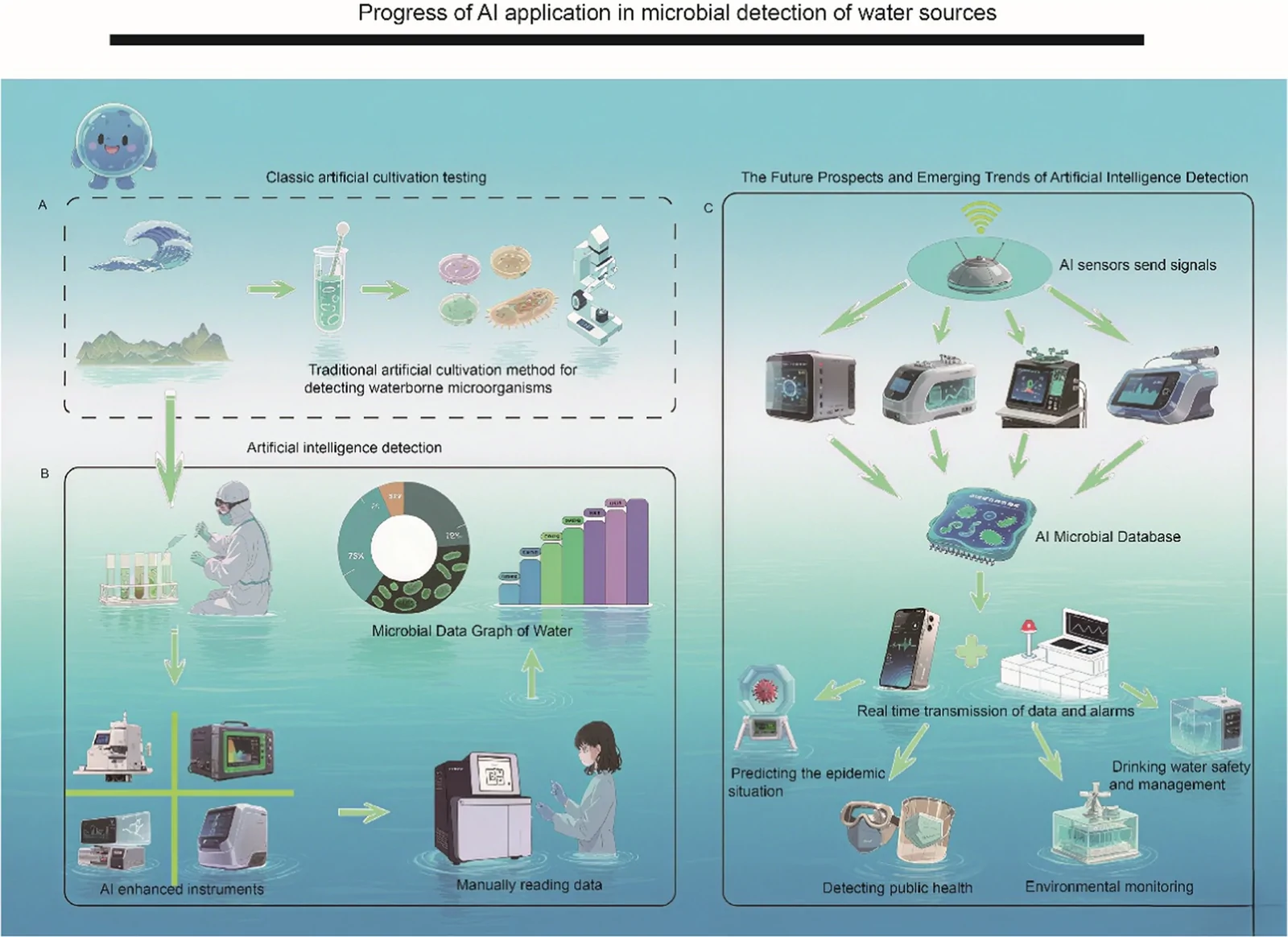
Traditional method for detecting microorganisms in water sources. **A** A classic approach that lays the foundation; Modern microbial detection methods for water sources. **B** Accurate and efficient technological innovation; The future prospects of water-based microbial detection. **C** Integrated innovation of intelligence and full chain prevention and control

AI technologies dominate the emerging evolutionary trend of aquatic microbial detection, facilitating the iterative upgrading of monitoring systems toward enhanced intelligence, autonomy, and systematic integration, as illustrated in Fig. 4.

First, the in-depth integration of AI and Internet of Things (IoT) technologies enables seamless interconnection among distributed sensors, terminal devices, and data centers, facilitating real-time acquisition and remote operational management of water quality parameters, and thereby significantly enhancing monitoring efficiency and operational intelligence levels. Second, multimodal data fusion assimilates heterogeneous information from diverse sources, including sensor readings, visual imagery, spectral datasets, and genomic sequencing data, to construct robust, high-precision, and holistic water quality assessment models, thereby strengthening the system’s early warning performance and pollution source traceability capacity. Third, federated learning serves as a privacy-preserving collaborative learning paradigm that enables multi-institutional joint model training without raw data disclosure. This approach safeguards data confidentiality while improving the generalization performance of models across diverse regional and scenario-specific conditions.

In rapid field detection scenarios, the integration of AI techniques with biosensors substantially boosts detection efficiency and analytical accuracy, driving the advancement of portable automated detection systems. Such systems exhibit prominent applicability for emergency early warning responses and resource-constrained monitoring environments. In the public health domain, AI leverages historical water quality, meteorological and epidemiological datasets to build disease risk prediction models, delivering scientific evidence for early intervention and preventive strategies against waterborne diseases.

Overall, AI-powered aquatic microbial detection is evolving toward real-time, intelligent, and systematic operational frameworks, offering robust technical underpinnings for aquatic ecological safety governance and public health safeguard. These convergent technological advancements collectively demonstrate that AI is undergoing a paradigm shift from a standalone analytical tool to an integrated intelligent ecosystem for aquatic microbial risk governance.

## Conclusion

This critical review systematically evaluates AI-enabled methodologies for aquatic microbial detection, covering image analysis, spectroscopy, genomics, and predictive modelling approaches. Comprehensive analysis yields the core key findings outlined below.

Under controlled laboratory conditions characterized by low-turbidity, sufficient annotated datasets, and standardized microbial culturing environments, AI-based methodologies deliver superior performance compared with conventional analytical approaches. Nevertheless, such performance merits are highly condition-dependent. In turbid aquatic matrices or scenarios with insufficient training datasets, the superior performance of AI techniques may be diminished or fully attenuated. These observations demonstrate that the efficacy of AI approaches is conditional rather than universally applicable, and the translation of laboratory-based AI achievements into routine practical applications remains in its infancy.

Across the four technical pathways, this study identifies prevalent inherent limitations constraining practical field deployment: (i) a significant discrepancy between laboratory-verified performance and on-site operational adaptability; (ii) The absence of standardized datasets and unified validation protocols results in low reproducibility of relevant research findings; (iii) AI models exhibit limited interpretability and degraded generalization capability across diverse complex aquatic matrices; (iv) insufficient in-situ validation based on field-collected heterogeneous samples.

Addressing the aforementioned technical gaps necessitates systematic and coordinated efforts: (1) constructing a unified multi-matrix database with standardized annotation specifications; (2) designing lightweight, interpretable models adaptable to field deployment scenarios; (3) formulating industry-recognized universal validation criteria; (4) advancing interdisciplinary collaboration among microbiologists, data analysts, and aquatic environmental practitioners.

In summary, AI offers promising technical solutions for aquatic microbial detection, yet its merits remain conditional, environment-specific and heavily reliant on dedicated evaluation benchmarks. Full exploitation of AI’s technological potential hinges not merely on incremental algorithmic innovation but more critically on systematic field validation, data standardization, and real-world-oriented performance calibration of detection systems.

## Data Availability

No data was used for the research described in the article.
